# Knockdown of ZNF268, which Is Transcriptionally Downregulated by GATA-1, Promotes Proliferation of K562 Cells

**DOI:** 10.1371/journal.pone.0029518

**Published:** 2012-01-03

**Authors:** Yan Zeng, Wei Wang, Jian Ma, Xianguo Wang, Mingxiong Guo, Wenxin Li

**Affiliations:** 1 State Key Laboratory of Virology, College of Life Sciences, Wuhan University, Wuhan, China; 2 Key Laboratory of the Ministry of Education for Plant Developmental Biology, College of Life Sciences, Wuhan University, Wuhan, China; Southern Illinois University School of Medicine, United States of America

## Abstract

The human *ZNF268* gene encodes a typical KRAB-C2H2 zinc finger protein that may participate in hematopoiesis and leukemogenesis. A recent microarray study revealed that *ZNF268* expression continuously decreases during erythropoiesis. However, the molecular mechanisms underlying regulation of *ZNF268* during hematopoiesis are not well understood. Here we found that GATA-1, a master regulator of erythropoiesis, repressed the promoter activity and transcription of *ZNF268*. Electrophoretic mobility shift assays and chromatin immunoprecipitation assays showed that GATA-1 directly bound to a GATA binding site in the *ZNF268* promoter *in vitro* and *in vivo*. Knockdown of *ZNF268* in K562 erythroleukemia cells with specific siRNA accelerated cellular proliferation, suppressed apoptosis, and reduced expression of erythroid-specific developmental markers. It also promoted growth of subcutaneous K562-derived tumors in nude mice. These results suggest that *ZNF268* is a crucial downstream target and effector of GATA-1. They also suggest the downregulation of *ZNF268* by GATA-1 is important in promoting the growth and suppressing the differentiation of K562 erythroleukemia cells.

## Introduction

The human *ZNF268* gene was cloned and characterized from an early human embryonic cDNA library [1]. Since that time, several alternative splice transcripts of *ZNF268* have been isolated [2], [3]. *ZNF268* encodes a typical KRAB-containing zinc finger protein [1]. A developmental expression study suggests that ZNF268 plays a role in the development of human fetal liver as well as the differentiation of blood cells [4]. Multiple lines of evidence support a role for ZNF268 in hematopoiesis and leukemogenesis. Krackhardt *et al.* identified KW-4, an alternative transcript of ZNF268, as one of the tumor-associated antigens in chronic lymphocytic leukemia [5]. A case survey by our group suggests that aberrant alternative splicing of *ZNF268* generates factors with prognostic value and contributes to human hematological malignancies [6]. Our previous studies have also shown that the human *ZNF268* promoter is atypical and that this promoter requires an intragenic element located within the first exon to mediate responses to cyclic-AMP response element binding protein (CREB) [7], [8], which can act as a proto-oncogene to regulate hematopoiesis and contribute to the leukemia phenotype [9]. We found that CREB-2 binds to the CREB-binding site within the minimal promoter region in the absence of Tax, the oncoprotein of human T-cell leukemia virus type 1 (HTLV-1), to enhance *ZNF268* promoter activity, while CREB-1 binds in presence of Tax to repress it [7], [8]. Use of microarrays to generate a transcriptional profile of human hematopoiesis during *in vitro* lineage-specific differentiation revealed that *ZNF268* expression continuously decreases during erythropoiesis [10]. We have recently found that the ZNF268 levels decline during specific differentiation of CD34+ cells to erythrocytes (data not shown). Taken together, these findings suggest that ZNF268 participates in development and differentiation associated with hematopoiesis, particularly erythropoiesis. However, how ZNF268 contributes to this process is unclear.

GATA-1, a member of the GATA family of zinc finger factors (GATA-1–GATA-6), plays an important role in gene regulation during hematopoiesis, including erythropoiesis [11], [12], [13], [14], [15]. Several advanced experimental approaches have revealed that GATA-1 is essential for the survival of erythroid progenitors as well as the terminal differentiation of erythroid cells, whereas GATA-2 is crucial for the maintenance and proliferation of immature hematopoietic progenitors [16], [17], [18]. GATA-1 was originally isolated as a factor that bound to the β-globin promoter and has subsequently been found to bind most, if not all, known erythroid genes [19]. GATA-1 plays critical roles in cell proliferation and terminal maturation associated with erythroid differentiation, which are uncoupled processes [20]. Many oncogene and tumor suppressor genes, such as those encoding c-myc, p53 and cyclin, reportedly participate in GATA-1-related erythropoiesis [19], [20], [21], [22], [23], [24], [25]. Although understanding how hematopoietic stem cells (HSCs) undergo lineage commitment and develop into various mature blood cells has been intensely investigated for many years, the network of regulation is still incompletely understood.

Here, we investigated the mechanism underlying decreased expression of ZNF268 as well as the consequences of ZNF268 downregulation in K562 cells, a human erythroleukemia cell line derived from a patient with chronic myelogenous leukemia [26], [27], [28]. We provide evidence that GATA-1 represses transcription of *ZNF268* and that *ZNF268* downregulation modulates growth and differentiation of K562 cells.

## Materials and Methods

### Generation of *ZNF268*-deficient cell lines

K562 cells (CCTCC, Wuhan, China) were seeded at a density of 1×10^5^ cells/ml in RPMI 1640 medium containing 10% fetal bovine serum (GIBCO), penicillin (100 U/ml), and streptomycin (100 µg/ml). Cells were maintained at 37°C in a 5% CO_2_ incubator. RNAi was constructed using pLLU2G plasmid, which was tagged with green fluorescent protein (GFP). Lentiviral particles containing short hairpin RNA (shRNA) targeted to ZNF268 mRNA and its control vector were purchased from Cyagen Biosciences (Guangzhou, China). K562 cells were transfected with ZNF268 shRNA lentiviral particles (lenti-shh-268) or particles containing vain plasmid (lenti-shh-control), which served as a negative control. The shRNA (shh-268) sequence used was 5′-TGC ACG CAT GGA AAG AGT TTG ATT CAA GAG ATC AAA CTC TTT CCA TGC GTG CTT TTT TC-3′. K562 cells were cultured in media containing recombinant lentiviral particles and 1 µg/ml polybrene for at least 48 h before being subjected to fluorescence-activated cell sorting (FACS).

### FACS and analysis

Approximately 1×10^5^ cells were collected and washed with PBS containing 1% BSA and 0.1% sodium azide. They were then incubated in the presence or absence of fluorochrome-conjugated antibodies against CD71 (BD Biosciences), glycophorin A, or mouse IgG (Biolegend). Cells transfected with lentivirus containing a GFP-encoding plasmid were analyzed and sorted at day 3, without undergoing any antibody treatment, to generate stably transfected cell lines. The cell cycle profile was analyzed by treating cells with 70% ethanol overnight at 4°C and staining them with propidium iodide (PI). Apoptosis was measured by staining cells with PI, as described above for cell cycle determination, or PE-conjugated Annexin V. FACS analysis was performed using a Beckman Coulter flow cytometer and EXPO32 software (Beckman).

### Xenograft model in nude mice

Animal experiments were performed under standard guidelines. The protocol was approved by the Committee on the Ethics of Animal Experiments of Wuhan University. The permit numbers of animal experiments for this study is SCXK 2009-0004. Approximately 1×10^7^ ZNF268-silenced or control K562 cells suspended in 200 µl serum-free RPMI 1640 medium were subcutaneously injected into the right flank of male BALB/c-nu athymic mice. Tumor-bearing mice were sacrificed 30 days later. Tumor masses were then excised, measured, and imaged. Expression of ZNF268 in the tumor tissues was analyzed by real-time polymerase chain reaction (PCR) and western blot.

### Electrophoretic mobility shift assays (EMSAs) and supershift assays

EMSAs were performed using the LightShift® Chemiluminescent EMSA Kit (Pierce) according to the manufacturer's instructions. Oligonucleotide probes used for EMSA are shown in Table 1. The sense and antisense strands were labeled with the Biotin 3′ End DNA Labeling Kit (Pierce) and annealed by step cooling from 90°C to room temperature. K562 nuclear extracts were obtained using the Cytoplasmic and Nuclear Protein Extraction Kit (Boster, Wuhan, China). Anti-GATA-1 antibody was purchased from Santa Cruz Biotechnology. For competition experiments, a 200-fold molar excess of unlabeled probe was added to the binding reaction just before the addition of the Bio-labeled probe. Reaction mixtures were fractionated on 6% nondenaturing polyacrylamide gels and transferred to positively charged nylon membranes (GE Healthcare) fixed by ultraviolet crosslinking. Mobility shift was detected using the Chemiluminescent Nucleic Acid Detection Module (Pierce).

**Table 1:** Oligonucleotides used in this study.

Oligonucleotide	Sequence (5′ to 3′)^a^	Location^b^
G1	AAAGAGATATTATCTTACATCAGTC	−1412 to −1388
G2	ATTACCATTTGATAAAGCAATCCTG	−611 to −587
G3	TGTTACTGAGTATCTACCCAAAGGA	−588 to −564
G4	CTCATATGTTTATCACAGCACTATT	−532 to −508
G5	TAGTGGCCACTATCTTCAGTGAAAC	−370 to −346
G6	AGTGTGGGATGATAGACAATGAAGA	−271 to −247
G7	AGAAAACTTGTATCTGCCTCTGTGA	−54 to −30
G8	AATCATGCGTGATAAAAGAATCCAT	+105 to +129
G9	CTAACAAAACTATCCCTTGTTCGAC	+206 to +230
G10	TGTTCGACTTGTATCTTTATATACT	+224 to +248
G11	TTGTTCCTCAGATAGCGTTCATCGC	+794 to +818
G1-m	AAAGAGATA**ggcgag**TACATCAGTC	
G1-s	ATATTATCTTACATCAGTCAC	−1406 to −1386
G1-a	AGCCAGGATGGTCTTAATCTC	−1266 to −1286
C-s	AATGGCGTGAACCCG	−1166 to −1152
C-a	GCAAACTCCCGACCTTA	−962 to −978
PES1^c^	GCATATAACGTACTATAGGGCG	−190 to −169
PE12^c^	CGTAACATCATGTATTGGCCAGTTGG	+104 to +79
PECS11^c^	ACCTGGCCAGGAAGGCCTGAG	+594 to +614
PECA^c^	TGAAGGGGCAGCAGAATAGA	+925 to +906
Uzf	TCATAAATGTGGCACGCATGC	
Lzf	GTTGCGATTTCTTATTGACGG	
GAPDH-s^d^	TGATGACATCAAGAAGGTGGTGAAG	
GAPDH-a^d^	TCCTTGGAGGCCATGTGGGCCAT	

a Underlined nucleotides represent GATA binding sites, and lowercase letters indicate mutated residues.

b Shown are the oligonucleotide positions, where +1 is the transcription start site of the *ZNF268* gene.

c Data are from Ref. [8].

d Data are from Ref. [8].

### Chromatin immunoprecipitation (ChIP)

ChIP assays were performed as described previously [7], [8]. Briefly, K562 cells were crosslinked with 1% formaldehyde at room temperature for 15 min. The cells were washed twice in phosphate buffered saline (PBS) and lysed in sodium dodecyl sulfate lysis buffer (Biyotime, Haimen, China). Chromatin fragments were prepared by sonicating lysates on ice. Lysates were then incubated with antibodies against GATA-1, FOG, CREB-2, TFIID, RNA polymerase II, or IgG (Santa Cruz Biotechnology, Inc., Santa Cruz, CA). Immunoprecipitated complexes were collected using protein A/G-agarose beads (Santa Cruz). The pellets were washed with dialysis buffer (2 mM EDTA and 50 mM Tris-HCl, pH 8.0) and incubated at 65°C for 4 h to reverse the formaldehyde cross-link. They were then digested with 20 mg/ml proteinase K (Biyotime) for 1 h. DNA was purified using the Cycle Pure Kit (Omega) and subjected to PCR amplification using primers (Table 1) for the promoter region containing the transcription factor binding site.

### Transient transfection and dual luciferase assay

Transient transfection and dual luciferase assays were performed as described previously [7], [8]. In brief, HEK293 and HeLa cells (CCTCC, Wuhan, China) were seeded in 48-well plates and transiently transfected using Lipofectamine 2000 (Invitrogen). Cells were co-transfected with promoter constructs based on pGL3-Basic (which expresses firefly luciferase from the putative *ZNF268* promoter) and the control construct pRL-TK (which expresses *Renilla* luciferase). Cells were harvested 48 h after transfection for dual luciferase assays (Promega).

### Western blotting

Cells were lysed with RIPA buffer (50 mM Tris-HCl, pH 7.5; 150 mM NaCl; 1% NP-40; 0.25% sodium deoxycholate). Equal amounts of extract were then electrophoresed on a 10% sodium dodecyl sulfate polyacrylamide gel electrophoresis gel and transferred to nitrocellulose filter membranes (Millipore). Membranes were immersed in blocking buffer (5% degreased milk powder) and incubated with antibodies against ZNF268, β-actin, c-myc (Santa Cruz Biotechnology), p53, cyclin-D1 (Cell Signaling Technology), or Flag (Sigma). They were then incubated with horseradish peroxidase-conjugated secondary antibodies (Pierce), and immunoreactivity was visualized using the SuperSignal chemiluminescent detection module (Pierce).

### Real-time quantitative PCR

Total RNA was reverse transcribed into cDNA using SuperScript II (Invitrogen, Carlsbad, CA, USA). Real-time quantitative PCR was performed using an ABI 7500 real-time PCR system (Applied Biosystems, Foster City, CA, USA) and the SYBR Green Real time PCR Mater Mix (TOYOBO, Osaka, Japan). Primers used for real-time quantitative PCR are shown in Table 1. Each PCR reaction was performed in triplex tubes, with glyceraldehyde 3-phosphate dehydrogenase (GAPDH) being used as an endogenous control to standardize the amount of sample RNA.

### Cell counting and EdU labeling

The effect of *ZNF268* silencing on K562 cell proliferation was tested by cell counting. Approximately 1×10^5^ cells were cultured in triplicate in 24-well plates. Cells were counted in a hemocytometer every other day. After day 3, half of the media were renewed daily. Proliferation was also estimated using the EdU incorporation assay. Briefly, cells (1×10^5^) were cultured in 24-well plates and exposed to 50 µM EdU (Ribobio, Guangzhou, China) for 4 h at 37°C. The cells were then fixed in 4% formaldehyde for 30 min at room temperature and permeabilized in 0.5% Triton X-100 for 10 min. Cells were washed with PBS, and each well was incubated with 400 µl 1XApollo® reaction cocktail for 30 min. DNA was then stained with 5 µg/ml Hoechst 33342 (200 µl per well) for 30 min and imaged under a fluorescent microscope.

### Statistical analysis

The data are expressed as the mean ± standard deviation from at least three separate experiments. The differences between groups were analyzed using the double-sided Student's *t* test, and a *p* value less than 0.05 was considered significant.

## Results

### GATA-1 represses *ZNF268* promoter activity and transcription

**Figure 1:**
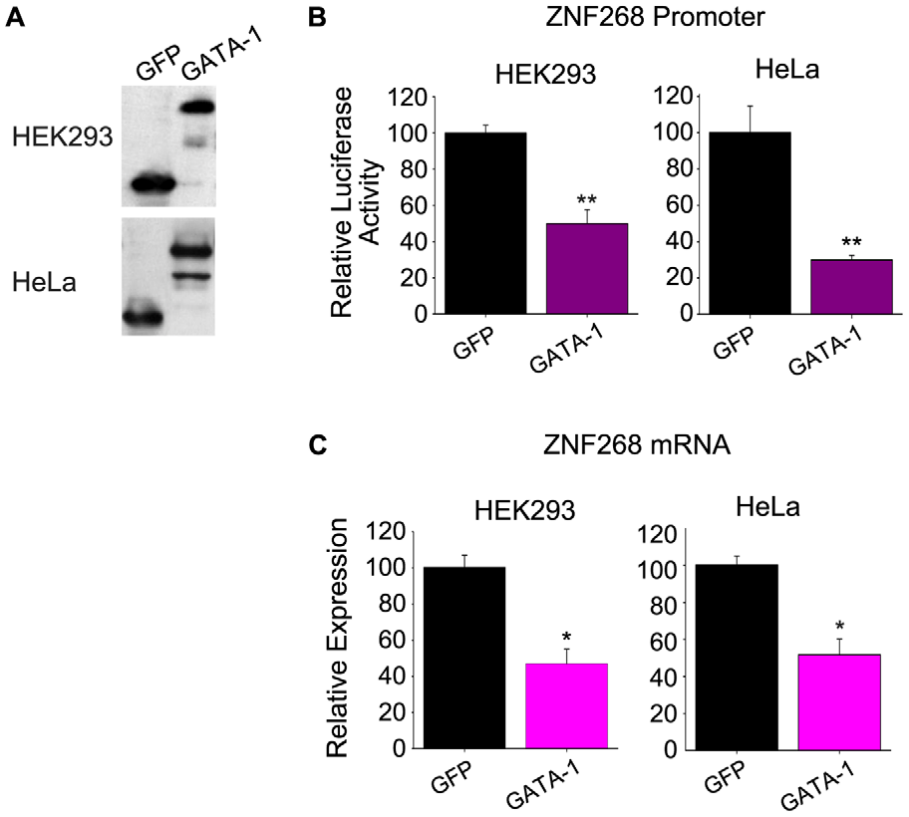
GATA-1 represses *ZNF268* promoter activity and transcription. (**A**) Western blot analysis of exogenous GATA-1 and GFP expression in transfected HEK293 and HeLa cells using anti-Flag antibody. (**B**) Luciferase assays in HEK293 and HeLa cells co-transfected with GATA-1 expression plasmid (0.2 µg in 48-well plates) and a luciferase reporter under the control of the *ZNF268* promoter. GFP expression plasmid served as a control. (**C**) Quantitative real-time PCR analysis of *ZNF268* mRNA in HEK293 and HeLa cells transfected with plasmid expressing GATA-1 or GFP. GAPDH mRNA was used to normalize *ZNF268* expression. Data (mean ± SD) are derived from an average of three independent experiments. *p<0.05 and **p<0.01 (standard *t* test).

Recent studies suggest that ZNF268 participates in human hematopoiesis, as seen by a decline in *ZNF268* expression during erythroid differentiation. To study how the *ZNF268* gene is regulated in hematopoietic cells, we searched for potential regulatory elements in the *ZNF268* promoter sequence. Using an online tool that predicts transcription factor binding sites (http://www.cbil.upenn.edu/cgi-bin/tess/tess), we identified 11 putative GATA binding sites within this region. GATA-1 has been long regarded as critical transcription factor for hematopoietic differentiation and is especially highly expressed during erythroipoiesis. To determine if GATA-1 regulates *ZNF268* promoter activity, we co-transfected HEK293 and HeLa cells with pCMV-3Tag-GATA-1 expression plasmid and a reporter plasmid carrying the luciferase gene under the control of the *ZNF268* promoter. Overexpression of GATA1 or GFP (negative control) in these cells was confirmed by western blot analysis using anti-Flag antibody (**Fig. 1A**). Analysis of luciferase activity in HEK293 and HeLa lysates revealed that GATA-1 overexpression significantly repressed the activity of the *ZNF268* promoter compared to GFP overexpression (**Fig. 1B**). We also measured *ZNF268* promoter activity in the presence of GATA-2, another founding member of the GATA family and a regulator of early stages of hematopoiesis [18]. We did not detect any changes in *ZNF268* promoter activity in the presence of GATA-2 overexpression (data not shown). Finally, we investigated whether transient overexpression of GATA-1 also affects the expression of *ZNF268* mRNA. Plasmids encoding GATA-1 or GFP were transfected into HEK293 and HeLa cells. Two days later, total RNA was isolated for quantitative real time PCR. As shown in **Fig. 1C**, GATA-1 overexpression reduced *ZNF268* mRNA by about one half.

### GATA-1 binds to a GATA binding site in the *ZNF268* promoter both *in vitro* and *in vivo*

**Figure 2:**
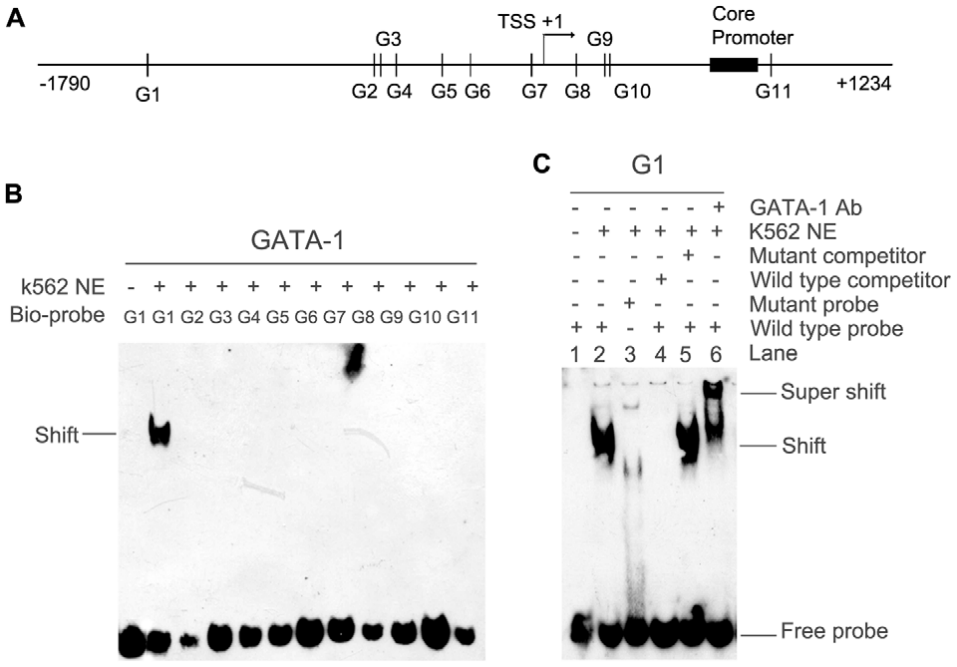
GATA-1 selectively binds to the GATA binding site in the *ZNF268* promoter *in vitro*. (**A**) Schematic diagram of the 11 GATA sites (G1–G11) in the *ZNF268* promoter. (**B**) EMSAs using K562 nuclear extract and biotin-labeled probes corresponding to the GATA binding sites in the human *ZNF268* promoter. Nuclear extract was omitted from the binding reaction as a negative control. (**C**) Competitive EMSAs and supershift assays showing the binding of a GATA-1 complex to the G1 site (−1412 to −1388). Labeled wild type G1 probe or labeled mutant probe was added to the reaction (*lanes 2* and *3*). Unlabeled competitors were added prior to G1 probe addition (*lanes 4* and *5*). For supershift experiments, anti-GATA-1 antibody was incubated with nuclear extracts before addition to the reaction mixture (*lane 6*).

To understand if GATA-1 interacts with putative GATA-1 elements in the *ZNF268* promoter (**Fig. 2A**), we first conducted EMSAs. EMSAs were performed using nuclear extracts from K562 cells and biotin-labeled double-stranded oligonucleotide probes containing sequences for the putative GATA-1 binding sites (Table 1). As shown in **Fig. 2B**, among the 11 probes (G1 to G11), only the biotin-labeled G1 probe formed a shifted band. Competitive EMSA assays were then conducted to further analyze specific binding to the G1 probe. We could detect no DNA/protein complex when the assay was repeated using a biotin-labeled mutant G1 probe (**Fig. 2C**, lane 3). Moreover, the complex formed from the biotin-labeled wild type G1 probe could effectively be competed away by the addition of 200-fold excess unlabeled wild type G1 probe (**Fig. 2C**, lane 4), but not by the same amount of unlabeled inactive mutant GATA-1 probe (**Fig. 2C**, lane 5). When anti-GATA-1 antibody was added to the reaction, a supershifted band appeared (**Fig. 2C**, lane 6). These results indicate that GATA-1 binds directly to the G1 binding site (−1412 to −1388 of the *ZNF268* promoter).

**Figure 3:**
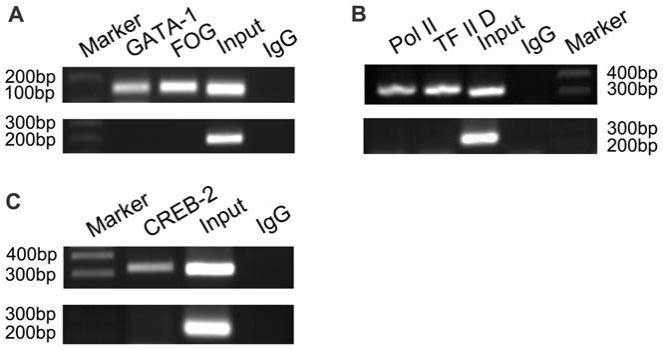
GATA-1 binds to the *ZNF268* promoter *in vivo*. ChIP assays were performed with K562 cells using the indicated antibodies, with IgG serving as a negative control. The precipitated DNA was amplified by PCR, electrophoresed, and stained with ethidium bromide. For all antibodies, primers C-s/C-a (−1166 to −962) served as a negative control. Input lanes show products after PCR amplification and before immunoprecipitation. (**A**) PCR amplification of DNA precipitated with anti-GATA-1 or anti-FOG antibodies using the primers G1-s/G1-a, which flank the GATA-binding sites contained within −1406 to −1266. (**B**) PCR amplification of DNA precipitated with anti-RNA polymerase II (pol II) or anti-TFIID antibodies using primers flanking the transcription start site (PES1/PE12). (**C**) As a positive control, ChIP assays were conducted using anti-CREB-2 antibody and primers flanking the CRE binding site in the *ZNF268* promoter (+594 to +925).

To understand whether the putative GATA-1 binding sequences in the *ZNF268* promoter can recruit GATA-1 to the promoter *in vivo*, we conducted ChIP assays. Chromatin fragments were prepared from K562 cells and immunoprecipitated with specific monoclonal antibodies to GATA-1 or FOG, which is an interacting partner of GATA-1 that has no sequence binding activity [29]. The isolated DNA was amplified by PCR with primers G1-s/G1-a (−1406 to −1266) (Table 1), which are specific for the promoter region containing the G1 site. When anti-GATA-1 or anti-FOG antibodies were used for the ChIP assay, a fragment of the expected size of 141 bp was detected (**Fig. 3A**). However, no signal was detected when anti-IgG antibody was used. A signal also failed to be detected when PCR amplification of the precipitated DNA was performed using primers specific for a region lacking a GATA-1 site (primers C-s/C-a, −1166 to −962; **Table 1** and **Fig. 3A**). Importantly, our system was validated by conducting ChIP assays using antibodies against TFII D or RNA polymerase II and primers flanking the transcription start site (**Fig. 3B**). Furthermore, as a positive control, assays were repeated using antibody to CREB-2, a known activator of the *ZNF268* promoter, and primers flanking the CRE binding site in the *ZNF268* promoter (**Fig. 3C**) [8]. Together, these ChIP assay results show that the transcription factor GATA-1 directly binds to the G1 binding site to form a complex in the *ZNF268* promoter *in vivo*.

### *ZNF268* silencing accelerates proliferation of K562 cells

**Figure 4:**
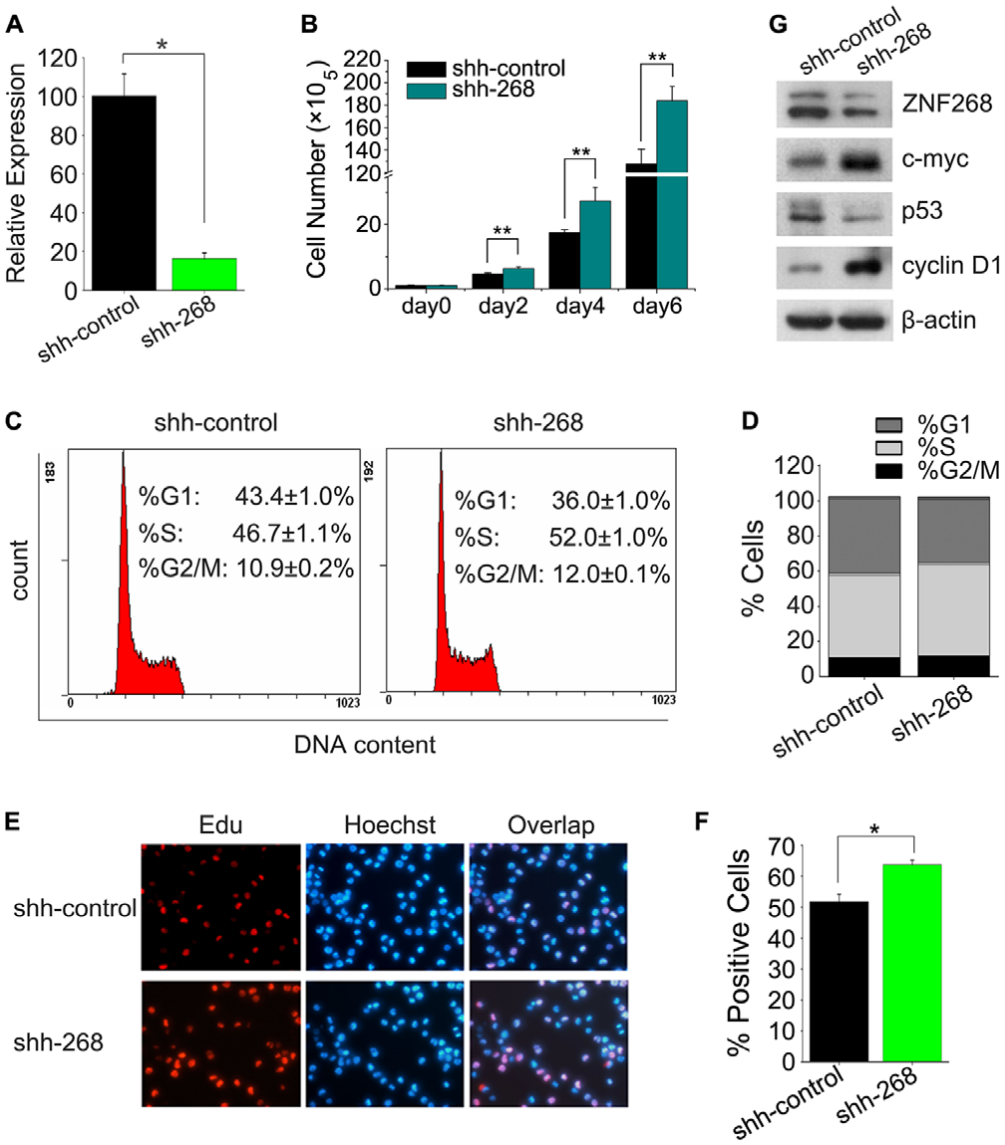
Stable silencing of *ZNF268* accelerates the proliferation of K562 cells. K562 cells were transfected with recombinant lentiviral particles containing ZNF268 short hairpin RNA (shRNA; shh-268) or control lentiviral vector (shh-control). Transfected cells were then sorted according to GFP expression to generate stably transfected cell lines. (**A**) Quantitative real-time PCR analysis of *ZNF268* mRNA in *ZNF268*-silenced and control cells. (**B**) Cellular proliferation, as determined by cell counting. Values are derived from an average of three independent experiments. (**C**, **D**) Cell cycle profiles, as assessed by DNA content in PI-stained cells. (**E**, **F**) EdU labeling showing proliferation of *ZNF268*-silenced and control cells. The percentage of positive cells was derived from triplicate samples. (**G**) Western blot analysis of c-myc, p53, and cyclin D1. ZNF268 and β-actin levels were also analyzed. *p<0.05 and **p<0.01 (standard *t* test).

To explore the consequences of GATA-1-mediated downregulation of *ZNF268* in K562 cells, we stably silenced *ZNF268* in K562 cells using recombinant lentiviral particles containing ZNF268 shRNAs. These cells exhibited significantly reduced expression of ZNF268 at both the mRNA and protein levels compared to control lentiviral vector-infected cells (**Fig. 4A, G**). As shown in **Fig. 4B**, stable silencing of *ZNF268* dramatically accelerated K562 cell proliferation, with the number of cells being 44% higher in the *ZNF268-*silenced group than the control group at day 6. Accordingly, FACS analysis of PI-stained cells revealed that the portion of cells in S phase was increased by approximately 5% and the proportion of cells in G1 phase decreased by a comparable degree (**Fig. 4C, D**). The effect of *ZNF268* silencing on proliferation was also measured using the EdU incorporation assay, which is more sensitive than cell counting. As anticipated, the number of EdU+ cells was approximately 10% higher in *ZNF268*-silenced cells than in control cells (**Fig. 4E, F**). These independent lines of data indicate that *ZNF268* silencing accelerates K562 proliferation *in vitro*.

To investigate the potential mechanisms underlying the ability of ZNF268 to influence K562 proliferation, we measured the relative levels of c-myc, p53, and cyclin D1 by western blot, with β-actin serving as an internal control. We found that *ZNF268* silencing upregulated c-myc and cyclin D1, while it downregulated p53 (**Fig. 4G**). This suggests that these molecules and their related networks may take part in ZNF268 regulation of K562 proliferation.

### *ZNF268* silencing suppresses apoptosis and promotes tumor growth *in vivo*

**Figure 5:**
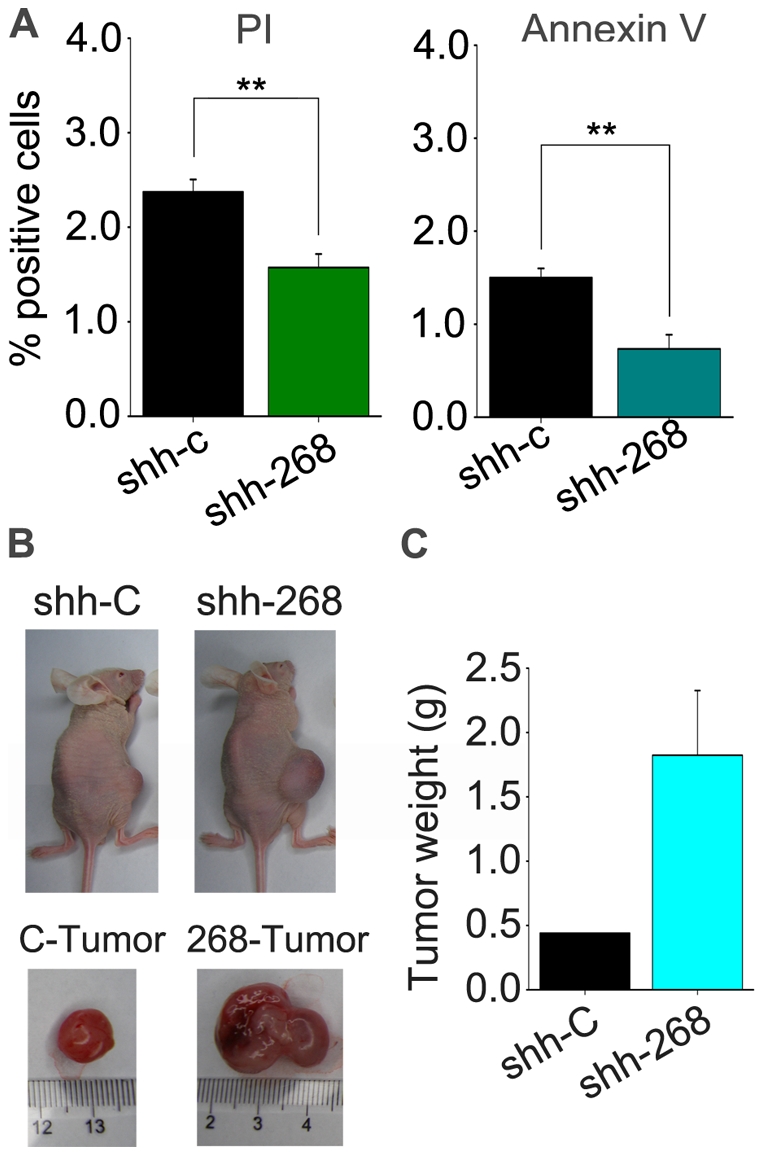
Stable silencing of *ZNF268* suppresses apoptosis and promotes tumor formation in nude mice. (**A**) Apoptosis in of *ZNF268*-silenced (shh-268) and control (shh-c) cells, as assessed by FACS analysis of PI or Annexin V staining. **p<0.01 (standard *t* test). (**B**, **C**). *ZNF268*-silenced or control K562 cells (∼1×10^7^ cells) were subcutaneously implanted into male athymic nude mice. Tumor-bearing mice were then sacrificed 30 days later. Representative mice and excised tumors are shown (**B**), along with a comparison of tumor weight between the groups (**C**).

We investigated the effect of stable *ZN268* silencing on not only K562 proliferation, but also apoptosis. Apoptosis was measured through FACS analysis of PI- or Annexin V-stained cells. As shown in **Fig. 5A**, *ZNF268* silencing induced a 34% to 51% decrease in basal apoptosis.

Next, we investigated the effect of *ZNF268* silencing *in vivo* by examining the tumorigenicity of K562 cells in nude mice. Mice received a subcutaneous injection of *ZNF268*-silenced clones (n = 5) or vector control clones (n = 5) in the right flank, so that tumor comparisons would be controlled for each individual mouse. Growth of tumors was monitored every 3 days, and tumors were excised and weighed 30 days after injection. We found that *ZNF268* silencing promoted subcutaneous tumor growth in nude mice (**Fig. 5B, C**). Quantitative real time PCR and western blot analysis of the tumors showed that ZN268 was suppressed in tumors formed from *ZNF268*-silenced K562 cells (data not shown).

### *ZNF268* silencing represses erythroid marker expression in K562 cells

**Figure 6:**
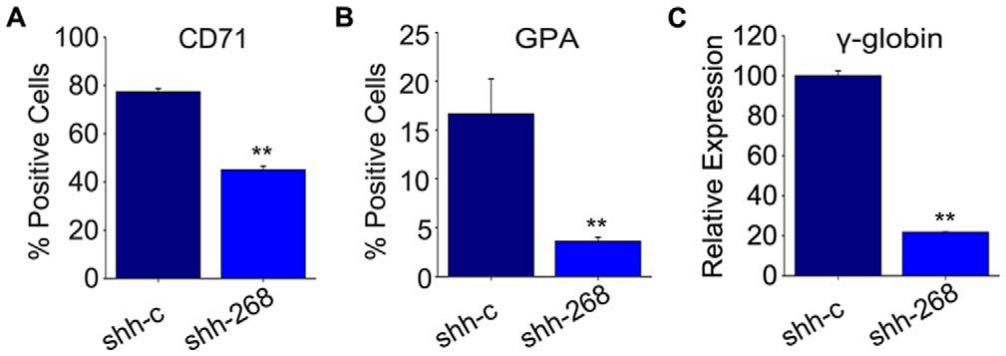
Stable silencing of *ZNF268* suppresses erythroid differentiation of K562 cells. (**A**, **B**) Surface expression of the erythroid markers CD71 and glycophorin A in *ZNF268*-silenced (shh-268) and control (shh-c) cells. Expression was analyzed using PE-conjugated antibodies against human CD71 or glycophorin A. PE-conjugated control IgG served as a negative control. (**C**) Quantitative real-time PCR analysis of γ-hemoglobin (γ-globin) mRNA in *ZNF268*-silenced and control cells. Expression of γ-globin was normalized to GAPDH. Data were derived from triplicate samples. **p<0.01 (standard *t* test).

Finally, we determined whether *ZNF268* silencing affects the differentiation of K562 cells. First, we examined erythroid differentiation by analyzing expression of CD71 and glycophorin A (CD235a), which are expressed during erythropoiesis. CD71 is expressed at the BFU-E stage and disappears at the late reticulocyte stage [30], [31], [32]. Glycophorin A first appears on the surfaces of proerythroblasts and is increasingly expressed during erythropoiesis [30], [31]. We found that the surface expression of these erythroid cell markers was lower in *ZNF268*-silenced cells than in control cells (**Fig. 6A, B**). We also analyzed the expression of γ-hemoglobin, which is regarded as an endogenous erythroid differentiation marker in K562 cells [33], [34]. Real-time PCR revealed that γ-hemoglobin mRNA levels in *ZNF268*-silenced cells were only one fifth of those in vector control cells (**Fig. 6C**). These results indicate that silencing *ZNF268* expression suppresses erythroid differentiation of K562 cells.

## Discussion

In this study, we have uncovered a possible mechanism by which *ZNF268* is repressed during erythroid differentiation. We have also characterized some of the effects of ZNF268 silencing in human K562 erythroleukemia cells. First, we have proven that GATA-1 downregulates the transcription of *ZNF268* by directly binding to a GATA binding site in the *ZNF268* promoter. The finding that a positive signal was detected using either an anti-GATA-1 antibody or anti-FOG antibody in ChIP assays suggests that the repressive action of GATA-1 on the *ZNF268* promoter is likely associated with FOG in K562 cells.

We have previously identified and studied the function of the *ZN268* promoter using deletion analyses [8]. These analyses suggested that the critical activated elements in the *ZNF268* promoter are located between −37 and +938, primarily after the transcription start site. Furthermore, CREB-2 was found to bind to the region spanning +589 to +760 and to strongly activate the *ZNF268* promoter. However, the upstream promoter region was found to have much lower activity, and this region was not studied any further. Here we demonstrate that the *ZNF268* promoter region from −1412 to −1388 binds to GATA-1 and represses *ZNF268* promoter activity as seen by luciferase activity assays. These findings suggest that the upstream region of the *ZNF268* promoter probably harbors elements for transcription factors that are repressive to *ZNF268*.

As already mentioned, we identified 11 putative GATA binding sites scattered throughout the promoter region of *ZNF268*. EMSAs revealed that GATA-1 bound to only the first site, which was the most distant and was located about 1.4 kb upstream from the *ZNF268* transcription start site. This binding was verified using the ChIP assay. A similar result has previously been reported in a study of GATA-1-dependent transcriptional repression of the *GATA-2* gene. GATA-1 reportedly binds to a highly restricted upstream region of the ∼70-kb GATA-2 domain, despite the presence of more than 80 GATA sites throughout the domain [35].

GATA-1 is an important regulator of erythropoiesis. This transcription factor binds to almost all known erythroid-related genes and takes part in erythroid differentiation at the level of cell proliferation and terminal maturation [19], [20]. To mimic the function of *ZNF268* repression by GATA-1, we used shRNA interference to stably silence *ZNF268* in K562 cells. We have provided the first evidence that *ZNF268* silencing promotes the proliferation of K562 cells and suppresses apoptosis and erythroid differentiation of these cells. These findings suggest that ZNF268 may function as a repressor of tumor cell proliferation. ZNF268 may be similar to Egr1, which has been identified as a target of GATA-1 by ChIP-seq analysis and is regarded as a tumor repressor [36], [37], [38].

In addition, our data suggest that *ZNF268* knockdown represses the erythroid differentiation of K562 cells. However, it seems not consistent with the observation that ZNF268 levels decrease continuously during specific differentiation of CD34+ cells to erthrocytes. Acturally, K562, as an erythroleukemia cell line, fail to mimic every features of normal human CD34+ cell differentiation into red cells. We believe that further investigation on the regulation network of *ZNF268* may contribute to understand human erythroid differentiation.
